# A novel genus of Pectobacterium bacteriophages display broad host range by targeting several species of Danish soft rot isolates

**DOI:** 10.1016/j.virusres.2024.199435

**Published:** 2024-07-16

**Authors:** Julie Stenberg Pedersen, Alexander Byth Carstens, Magnus Mulbjerg Rothgard, Chayan Roy, Anouk Viry, Bhavya Papudeshi, Witold Kot, Frank Hille, Charles M.A.P. Franz, Robert Edwards, Lars Hestbjerg Hansen

**Affiliations:** a Department of Plant and Environmental Sciences, University of Copenhagen, Thorvaldsensvej 40, Frederiksberg 1871, Denmark; b Department of Microbiology and Biotechnology, Max Rubner-Institute, Hermann-Weigmann-Str. 1, 24103 Kiel, Germany; c Flinders Accelerator for Microbiome Exploration, College of Science and Engineering, Flinders University, Adelaide, Australia

**Keywords:** Phages, Soft rot *Pectobacteriaceae*, Biocontrol, Genome analysis, Phage-host interactions

## Abstract

The bacterial diseases black leg and soft rot in potatoes cause heavy losses of potatoes worldwide. Bacteria within the genus *Pectobacteriaceae* are the causative agents of black leg and soft rot. The use of antibiotics in agriculture is heavily regulated and no other effective treatment currently exists, but bacteriophages (phages) have shown promise as potential biocontrol agents. In this study we isolated soft rot bacteria from potato tubers and plant tissue displaying soft rot or black leg symptoms collected in Danish fields. We then used the isolated bacterial strains as hosts for phage isolation. Using organic waste, we isolated phages targeting different species within *Pectobacterium*. Here we focus on seven of these phages representing a new genus primarily targeting *P. brasiliense*; phage Ymer, Amona, Sabo, Abuela, Koroua, Taid and Pappous. TEM image of phage Ymer showed siphovirus morphotype, and the proposed Ymer genus belongs to the class *Caudoviricetes,* with double-stranded DNA genomes varying from 39 kb to 43 kb. *In silico* host range prediction using a CRISPR-Cas spacer database suggested both *P. brasiliense, P. polaris* and *P. versatile* as natural hosts for phages within the proposed Ymer genus. A following host range experiment, using 47 bacterial isolates from Danish tubers and plants symptomatic with soft rot or black leg disease verified the *in silico* host range prediction, as the genus as a group were able to infect all three *Pectobacterium* species. Phages did, however, primarily target *P. brasiliense* isolates and displayed differences in host range even within the species level. Two of the phages were able to infect two or more *Pectobacterium* species. Despite no nucleotide similarity with any phages in the NCBI database, the proposed Ymer genus did share some similarity at the protein level, as well as gene synteny, with currently known phages. None of the phages encoded integrases or other genes typically associated with lysogeny. Similarly, no virulence factors nor antimicrobial resistance genes were found, and combined with their ability to infect several soft rot-causing *Pectobacterium* species from Danish fields, demonstrates their potential as biocontrol agents against soft rot and black leg diseases in potatoes.

## Highlights

• Phages present in the Ymer genus represent a new genus.
• An *in-silico* host range prediction indicated phages to infect broadly.
• The *in-silico* host range potential was verified experimentally.
• Phages were able to infect several species of soft rot *Pectobacteriacea*.
• The Ymer genus represents important biocontrol agents against soft rot.

## Graphical abstract

**Figure:**
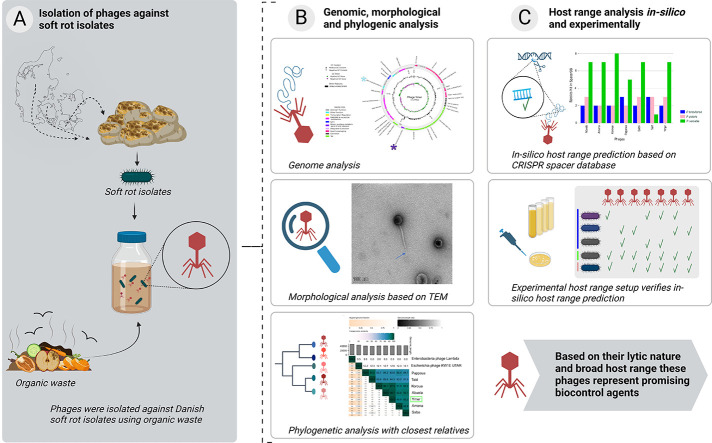

## 1 Introduction

Soft rot *Pectobacteriaceae* (SRP) includes the two genera *Pectobacterium* and *Dickeya*. These pathogens can cause disease in a wide variety of vegetables and ornamental plants worldwide. Their pectinolytic activity causes tissue maceration leading to water-soaked lesions, wilting and death of plants (Charkowski, 2018; Oulghazi et al., 2021). SRP causes soft rot in potato tubers and blackleg in potato plants. Soft rot and black leg are among the economically most important diseases of potatoes. SRP influence both seed production affecting seed certification, yield of harvest and even post-harvest loss due to soft rot during storage (Dupuis et al., 2021). In Denmark the traditional cloned multiplication system was abandoned in 1977 when the Danish Meristem Programme was implemented, to ensure completely disease-free material in the production of seed tubers (Ministry of Food, 2014). Nevertheless, Danish plant consultants and potato farmers have seen a rise in incidences of soft rot and blackleg during the last 10 years without any known cause (personal communication with Danish plant consultants and potato farmers). Disease management mainly relies on trying to avoid contamination of the pathogen by different methods, both by using seed certification systems, focusing on a good hygiene practice when handling equipment and during harvest, field inspections during the growth season, as well as using low temperatures and low humidity during storage. Both physical (hot water treatment) and chemical treatment have been tried but with limited success (Czajkowski et al., 2011). Also, breeding for resistance has not yet succeeded (Chung et al., 2017). Another potential way to manage the disease could be the use of biocontrol agents. The main biocontrol strategies against soft rot which have been tested are the use of bacterial antagonist's, quorum-quenching bacteria and natural compounds which may affect the pathogen population by different mechanisms. Another strategy is the use of bacteriophages (phages), which are natural viruses of bacteria (Hadizadeh et al., 2019). Several studies have isolated and described phages targeting SRP (Carstens et al., 2018; Carstens et al., 2019; Djurhuus et al., 2020; Muturi et al., 2019; Pedersen et al., 2020; Zaczek-Moczydłowska et al., 2020), and some of these have further evaluated the isolated phages as biocontrol agents. Carstens et al. showed that both disease incidence and severity decreased significantly when applying a phage cocktail in a tuber assay performed under simulating storage conditions, using *P. atrosepticum* (Carstens et al., 2019). Zaczek-Moczydlowska et al. also demonstrated that phages can decrease tissue maceration in a tuber assay using both *P. atrosepticum* and *P. carotovorum* subsp. *carotovorum*. They further showed that when phages were applied in vivo in a field experiment, they would get a higher emergence of sprouts as well as a higher yield when compared to their negative control (Zaczek-Moczydłowska et al., 2020). These studies highlight the potential of phages as part of a new era of disease management in agriculture. In this study we aimed to isolate and characterize phages being able to infect Danish bacterial soft rot isolates. We used Danish bacterial isolates from symptomatic plants and tubers, collected during 2021, to isolate phages targeting different species within *Pectobacterium*. Here we present a new genus of seven of these phages, the Ymer genus which, based on nucleotide similarity, currently do not have any close relatives with any viruses present at the GenBank database.

## 2 Materials and methods

### 2.1 Isolation of bacteria

Bacteria were isolated from diseased tubers and potato plants showing symptoms of soft rot and black leg, send by Danish potato producers in the period April to December 2021. The isolation method was adapted from Zaczek-Moczydłowska et al. (2019), using crystal violet pectin single layer (CVP), described by Hélias et al. (2012), for isolation of soft rot *Pectobacteriaceae*. Samples were taken with a sterile loop inside the macerated tissue in the plant or tuber and diluted in 1.6 ml sterile water. The samples were vortexed for 30 s, incubated for at least 5 min at RT and vortexed again for 30 s. 100 µl of the plant material extract were plated on CVP plates using a Drigalski spatula, the same spatula was then used for 4 more plates without sample fluid, to dilute the sample. CVP plates were incubated at 28 °C for 48 h. Cavity forming colonies were re-streaked on CVP plates and incubated at 28 °C for 48 h. Single cavity forming colonies were then streaked on Lysogeny Broth (LB) with 1.5 % agar and incubated for 1–2 days at 28 °C. Purified bacterial colonies were inoculated in 10 ml LB and incubated overnight (ON) at 28 °C. 15 % glycerol stocks were prepared using ON cultures and stored at −80 °C for further use.

### 2.2 Isolation and purification of phages

Phages were isolated from an organic waste sample as described elsewhere (Carstens et al., 2019). Prior to isolation the organic waste sample was centrifuged at 10,000 × *g* for 10 min at 4 °C and filtered through a 0.45 µm PVDF syringe filter (Fisherbrand™) before use. All hosts are listed in suppl. Table 1 and were all grown in LB liquid media at 28 °C ON prior to use. Phages were isolated using the standard double agar overlay (Kropinski et al., 2009) with 4 ml LB medium supplemented with 0.4 % agarose and 10 mM CaCl_2_·MgCl_2_, using 100 µl ON host (corresponding to ∼3 × 10^8^ CFU/ml) and 200 µl sample in the overlay agar. Plates were incubated at 28 °C ON. Afterwards plates were checked for plaque formation and plaques were picked using 1000 µl pipette tip and let to diffuse in 500 µl SM buffer (100 mM NaCl, 8 mM MgSO_4_·7H_2_O, 50 mM Tris-Cl pH 7.5) for at least 1 h. Phages were purified 3 times using the double layer overlay as described above. Purified phages were used to prepare high titer amplifications. Briefly, 100 µl of the purified phage stock in SM buffer was mixed with 100 µl of ON culture of isolation host in 10 ml LB medium supplemented with 10 mM CaCl_2_·MgCl_2_, and incubated shaking at 28 °C ON. The lysates were centrifuged at 10,000 × *g* for 10 min at 4 °C and filtered through a 0.45 µm PVDF syringe filter as described above.

### 2.3 DNA extraction and sequencing of bacteria and phages

Phage lysates (>10^6^ PFU/ml) were used for DNA extraction as described elsewhere (Carstens et al., 2018). The DNA was purified using DNA Clean & Concentrator™−5 (Zymo Research) according to manufacturer's protocol and DNA was eluted in 20 µl elution buffer. Sequencing libraries were prepared using NEBNext® Ultra™ II FS DNA Library Prep Kit for Illumina (New England Biolabs) according to manufacturer's protocol and libraries were sequenced with the NextSeq500 platform using the Mid Output Kit v2 (300 cycles), as previously described (Forero-Junco et al., 2022).

For bacterial DNA extraction, 1 ml of ON cultures was used as input for Genomic Mini AX Bacteria (A&A Biotechnology) kit following manufacturer's protocol and DNA was dissolved in sterile sigma water. Sequencing libraries were prepared for Illumina paired-end sequencing and Nanopore sequencing using the Nextera XT DNA Library Kit (Illumina) and the Rapid Barcoding Kit (Oxford Nanopore Technologies). Indexed DNA were both sequenced using Illumina NextSeq and using Nanopore Minion. To complete all genome assemblies, some of the genomes were re-sequenced using Nanopore Promethion.

### 2.4 Assembly and annotation of phage genomes

Raw reads from sequencing were used to assemble phage genomes using metaSPAdes v. 3.14.1 (Nurk et al., 2017). Start site was predicted by PhageTerm v. 4.1 (Garneau et al., 2017) for 2 out of 7 phages (Amona and Pappous), and aligned in the rest of the phages manually using CLC Genomics Workbench 22.0 (QIAGEN, Aarhus, Denmark). Thereafter, the CLC Genomics Workbench 22.0 was used to manually delete terminal redundant duplicates within the genome of all phages. All phages were annotated with Pharokka v. 1.7.0 (Bouras et al., 2023). Specifically, coding sequences (CDS) were predicted with PHANOTATE (McNair et al., 2019), transfer RNAs (tRNAs) were predicted with tRNAscan-SE 2.0 (Chan et al., 2021), transfer-messenger RNAs (tmRNAs) were predicted with Aragorn (Laslett and Canback, 2004) and Clustered Regularly Interspaced Short Palindromic Repeats (CRISPRs) were predicted with CRT (Bland et al., 2007). Functional annotation was generated by matching each CDS to the PHROGs (Terzian et al., 2021), VFDB (Chen et al., 2005) and CARD (Alcock et al., 2020) databases using MMseqs2 (Steinegger and Söding, 2017) and PyHMMER (Larralde and Zeller, 2023). Contigs were matched to their closest hit in the INPHARED database (Cook et al., 2021) using mash (Ondov et al., 2016). BLAST was used to search for genome similarity (Sayers et al., 2022). Plots were created with pyCirclize (Shimoyama, 2022). The large terminase was manually annotated in all phage genomes based on gene synteny and amino acid sequence similarity with terminases, using blastp against the nr protein database (Sayers et al., 2022).

### 2.5 Assembly and classification of bacterial genomes

Raw reads from both Illumina and Nanopore for all bacterial isolates were used for assembly using Unicycler version 0.5.0 (Wick et al., 2017). ContEST16S (Lee et al., 2017) was used to extract 16S rRNA gene sequences for all bacterial genomes. Remaining genomic sequences will be assembled and published elsewhere. Species known to cause soft rot in potatoes (Charkowski, 2018; Curland et al., 2021) were used as reference genomes and were likewise used to extract 16S rRNA gene sequences using ContEST16S. Species included as reference genomes were as follows; *Dickeya dadantii* (RefSeq ID: NC_014500.1), *Dickeya dianthicola* (RefSeq ID: NZ_CP031560.1), *Dickeya solani* (RefSeq ID: NZ_CP017454.1), *Dickeya chrysanthemi* (RefSeq ID: NC_012912.1), *P. parmentieri* (RefSeq ID: NZ_CP027260.1), *P. atrosepticum* (RefSeq ID: NZ_CP009125.1), *P. aroidearum* (RefSeq ID: NZ_CP065044.1), *P. punjabense* (RefSeq ID: NZ_CP038498.1), *P. carotovorum* (RefSeq ID: NZ_CP051652.1), *P. versatile* (RefSeq ID: NZ_CP021894.1), *P. polaris* (RefSeq ID: NZ_CP017482.1), *P. brasiliense* (RefSeq ID: NZ_CP047495.1). Phylogeny.fr (Dereeper et al., 2008) was used for 16S rRNA gene-based phylogenetic analysis, using MUSCLE (Edgar, 2004) for multiple alignment, Gblocks (Castresana, 2000) for automatic alignment curation, PhyML (Guindon and Gascuel, 2003) for tree building and TreeDyn (Chevenet et al., 2006) for tree drawing. ITOL (Letunic and Bork, 2021) was used for tree annotation afterwards. Based on closest relative, bacterial isolates were divided into the following species; *Dickeya solani, P. parmentieri, P. atrosepticum, P. punjabense, P. versatile, P. polaris* and *P. brasiliense* (suppl. fig. 2). Bacterial isolates which did not classify into any species within reference sequences based on the 16S phylogenetic analysis were excluded in this study and not included in the host-range experiment (data not shown).

### 2.6 Comparative genomics

Phylogenetic analyses were carried out for conserved phage proteins (large terminase and major head protein). Based on blastp search using large terminase and major head protein in phage Ymer, the five closest relatives were included for each protein as reference genomes. Protein sequences for large terminase and major head protein from each phage was used for phylogenetic analysis. Phages included in the phylogenetic analysis were as follows; Escherichia phage KW1E UTAR (acc. no. MZ506873), Serratia phage vB SmaM-Susuwatari (acc. no. ON287371), Serratia phage vB SmaM-ChibiTotoro (acc. no. ON287368), Klebsiella phage P85_2 (acc. no. OR256025), Klebsiella phage vB Kpn K34PH164 (acc. no. OY979427), Escherichia phage PC2 (acc. no. NC_073088), Enterobacteria phage EK99P-1 (acc. no. NC_024783), Escherichia phage vB EcoD Teewinot (acc. no. NC_073053), Escherichia phage slur05 (acc. no. NC_028901), Escherichia phage vB_EcoS-EE09 (acc. no. OR756193). Furthermore, did we include Enterobacteria phage Lambda (acc. no. NC_001416.1) as reference genome. Phylogeny.fr (Dereeper et al., 2008) was used for phylogenetic analysis based on conserved phage proteins, using MUSCLE (Edgar, 2004) for multiple alignment, Gblocks (Castresana, 2000) for automatic alignment curation, PhyML (Guindon and Gascuel, 2003) for tree building and TreeDyn (Chevenet et al., 2006) for tree drawing. ITOL (Letunic and Bork, 2021) was used for tree annotation afterwards. Intergenomic similarity score was calculated and visualized using VIRIDIC (Moraru et al., 2020). Comparative genomics of all phages were done using Clinker v.0.0.28 (Gilchrist and Chooi, 2021). Enterobacter phage Lambda (accession number: NC_001416.1) and Escherichia phage KW1E UTAR (accession number: MZ506873.1) were both included in the clinker comparison. ColabFold version 1.4.0 were used to predict the secondary protein structure of two phage proteins annotated as membrane-associated proteins (Mirdita et al., 2022). PyMOL version 2.5.5 was used to visualize and align the two membrane-associated proteins (Schrödinger, 2023).

### 2.7 TEM analysis

CsCl gradient ultracentrifugation (Sambrook et al., 1989) was done to obtain clean high titer solution of phage Ymer prior TEM imaging. Morphological analysis on purified phage particles was conducted as described before (Riber et al., 2023). Briefly, cesium chloride-purified phages were pipetted on 100-mesh copper grids coated with carbon and adsorption was allowed for 20 min. Subsequently, the grids were washed twice with deionized water and negatively stained with 2 % uranyl acetate. Electron micrographs were generated on a Talos L120C transmission electron microscope using a 4 *k* × 4 k Ceta camera (both microscope and camera from ThermoFisher Scientific, Eindhoven, The Netherlands) set to an acceleration voltage of 80 kV. Phage head and tail sizes were measured using the TEM analysis software Velox v3.9.0 (ThermoFisher Scientific, Eindhoven, Netherlands) and the mean size and standard deviation of nine phage particles were calculated. The TEM images were manually improved using GIMP v2.10.32 for contrast increasing and brightness adjustment.

### 2.8 *In-silico* host prediction

To predict phage hosts species, we used the spacer database, SpacerDB, using CrisprOpenDB (Dion et al., 2021). BLAST+ ver. 1.14.1 (Camacho et al., 2009) was used to align isolated phage genomes to the spacer database. Data was visualized using Rstudio v. 4.3.1. (R Core Team, 2023). All spacers were mapped to phage genomes using the CLC Genomics Workbench 22.0 and visualized using a compiled consensus genome of the Ymer genus created with Clinker v.0.0.28 (Gilchrist and Chooi, 2021). Number of spacer sequences (length 20–50 bp) present in the database were calculated for all *Pectobacterium* species, known to be causing soft rot in potatoes (Charkowski, 2018; Curland et al., 2021), using CrisprOpenDB's web interface (http://crispr.genome.ulaval.ca/).

### 2.9 Host range experiment

To determine initial host-range, all seven phages were spotted on 47 bacterial isolates from potato tubers and plants symptomatic with soft rot and black leg (suppl. Table 1.). All strains were grown in LB at 28 °C ON. For the spot test, as well as the following host range experiment, the titer of all phages varied from 1.3 × 10^9^ to 5 × 10^9^ PFU/ml, except for phage Koroua which had a titer of 8.3 × 10^10^ PFU/ml. 10 µl of phage lysates were spotted on a bacterial lawn, using LB agarose (0.4 % agarose) supplemented with 10 mM CaCl_2_·MgCl_2_, with 100 µl of ON host bacteria. To evaluate any prophage activity of the phage hosts, all phage host ON cultures were centrifuge at 10,000 × *g* for 10 min at 4 °C and filtered through a 0.45 µm PVDF syringe (as described earlier) and likewise spotted on all 59 bacterial isolates. Plates were then incubated ON at 28 °C before the presence of a clearing zone was noted. All phage lysates (or filtered phage host) displaying lysis on a given host were further tested to determine the efficiency of plating (EOP) of that phage. Briefly, serial dilution of each phage or filtered phage host was spotted in triplicates of 10 µl on each susceptible host, as described above, to determine EOP. EOP was calculated as titer on the test host divided by titer on isolation host.

## 3 Results

### 3.1 A new branch of phages with very limited nucleotide sequence similarity to other phages in the NCBI database

The seven isolated *Pectobacterium* phages: Ymer, Amona, Sabo, Abuela, Koroua, Pappous and Taid are all dsDNA phages and have genome sizes ranging from 39,941 bp to 43,206 bp with very similar GC content of ∼46 % (Table 1). Less than half of their coding sequences (CDS's) could be assigned with a putative function (ranging from 42 to 49 %). When blasting the genome of phage Ymer (blastn; default parameters against the nr/nt database) the only hits obtained were with *Pectobacterium* and *Yersinia* bacterial strains with a query cover of 0–1 % and percent identity of ∼80 % and no phage hits. Most of the blast hits obtained for *Pectobacterium* and *Yersinia* bacterial strains were one hit within the gene encoding the minor tail protein, as well as one hit on the gene encoding a tRNA. Packaging strategy was predicted for two out of seven phages being categorized as headful packaging (Table 1, suppl. fig. 1), where the phage typically will create a concatemer with several copies, where the first cut during packing is made at a fixed point (*pac* site) which will lead to a peak in coverage of the sequencing reads, where the following cuts will be made when the head is full which will lead to various sites (no peak in sequencing reads coverage) (Garneau et al., 2017).

**Table 1:** Genomic information on the seven phages in the proposed Ymer genus inclusive host (see suppl. Table 1 for full description of all host), length, GC content (in phage and host), number of CDS, unknown CDS (hypothetical), tRNA, predicted packaging system by PhageTerm, as well as accession numbers for raw sequencing files in the SRA database.

**Phage**	**Host**	**Length**	**GC content**	**GC content in host**	**no. of CDS**	**Unknown function (Hypothetical CDS)**	**tRNA**	**Predicted packaging system**	**SRA accession no.**
Ymer	J36	41,279 bp.	46.38 %	52.03 %	64	33	1	–	SRX24744245
Amona	J25	43,206 bp.	46.33 %	52.03 %	68	36	1	Headful packaging	SRX24744247
Sabo	J24	42,167 bp.	46.20 %	52.03 %	64	32	1	–	SRX24744244
Abuela	J34	41,492 bp.	46.41 %	52.03 %	64	34	1	–	SRX24744246
Koroua	J52	41,346 bp.	46.44 %	52.03 %	64	33	1	–	SRX24744248
Pappous	J13	40,850 bp.	46.56 %	52.04 %	61	30	1	Headful packaging	SRX24744243
Taid	J31	39,941 bp.	46.80 %	51.99 %	60	30	1	–	SRX24744242

### 3.2 Taxonomical and morphological characterization

**Fig. 1:**
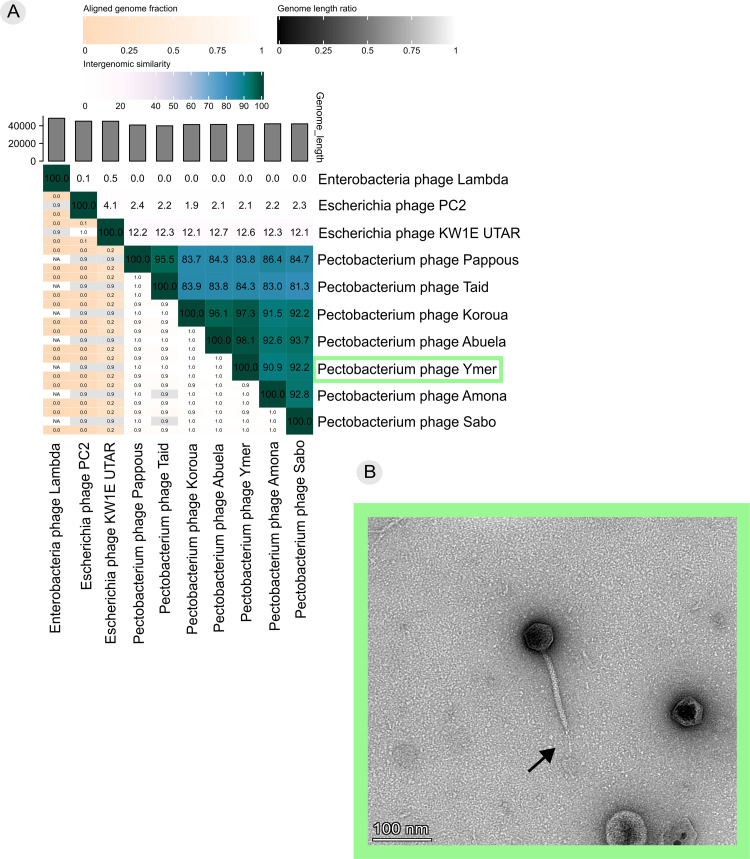
Taxonomical and morphological characterization. a) Intergenomic similarity score for all versus all phage genomes included closest relatives based on protein similarity with conserved phage proteins (large terminase and major head protein); Escherichia phage KW1E UTAR (acc. no. MZ506873.1) and Escherichia phage PC2 (acc. no. NC_073088). Additionally, did we also include Enterobacteria phage Lambda (acc. no. NC_001416.1). All phage genomes within the Ymer genus share >80 % intergenomic similarity representing a new genus. b) Transmission electron microscopy of phage Ymer showing siphovirus morphotype with central tail fiber (black arrow).

**Fig. 2:**
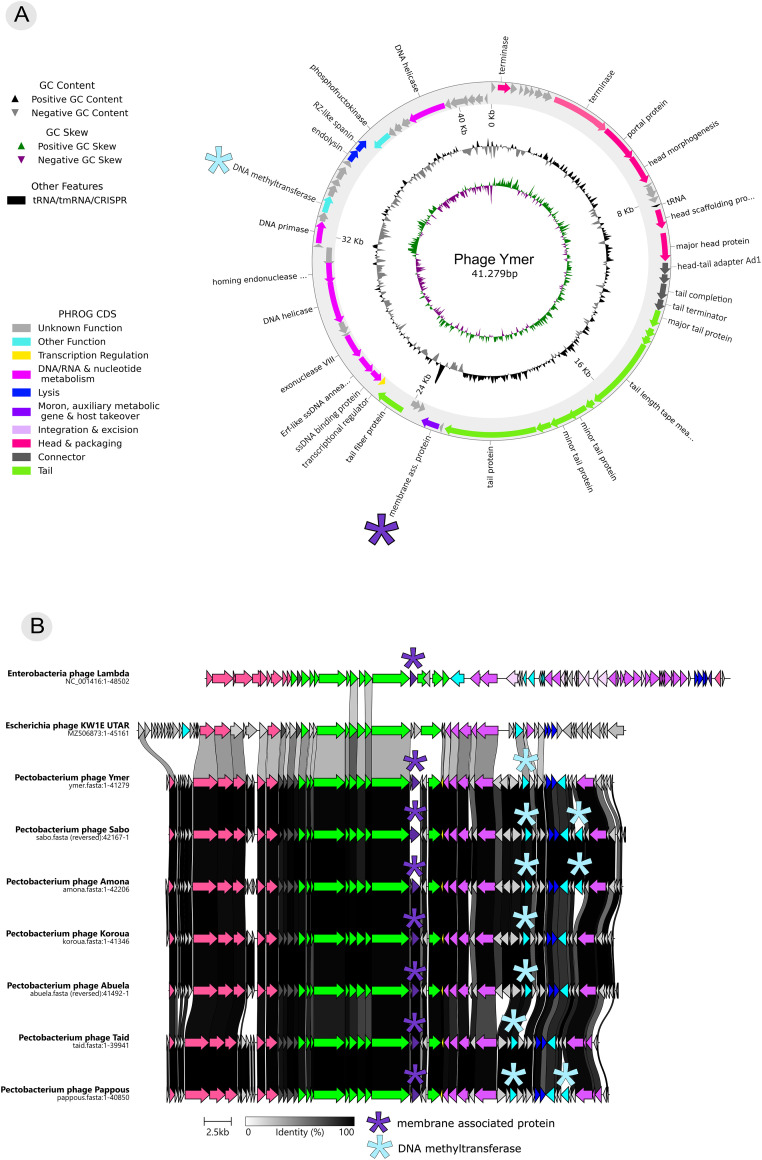
Genomic characterization of the Ymer genus. a) Genome visualization of the genome of phage Ymer. Annotated genes are colored based on their PHROG (Prokaryotic Virus Remote Homolog Groups): unknown function (grey), other function (turquoise), transcription regulation (yellow), DNA metabolism (light purple), lysis (dark blue), moron (dark purple), integration & excision (light rosa), head & packaging (pink), connector (dark grey), tail (green). Purple star represent gene annotated as membrane-associated protein, light blue star represent gene annotated as DNA methyltransferase. b) Genomic comparison of all phage genomes within the Ymer genus together with Enterobacter phage Lambda (acc. no. NC_001416.1) and Escherichia phage KW1E UTAR (acc. no. MZ506873.1). Annotated genes are colored based on their PHROG, purple stars represent genes annotated as membrane-associated proteins and light blue star represent genes annotated as DNA methyltransferases, as in a).

The seven phages constitute one genus, with an intergenomic similarity score of more than 80 % for all versus all (Fig. 1a) (Adriaenssens and Brister, 2017). No nucleotide similarity was found between the seven phages and other phages in the database (blastn; default parameters against the nr/nt database) and we propose the seven phages to represent a new genus, hereof described as the Ymer genus. The seven phages within the genus divides into four species with intergenomic similarity scores of more than 95 %, with phage Pappous and Taid forming one species, phage Koroua, Abuela and Ymer forming another species, and phage Amona and Sabo each being representative for two new species. As no nucleotide similarity was found between the Ymer genus and other phages, phylogenetic analyses, using protein sequence, based on conserved phage proteins (large terminase and major head protein) was used to find closest relatives (suppl. fig. 3). Based on the closest relative to the Ymer genus (using large terminase and major head protein based on blastp), Escherichia phage KW1E UTAR (acc. no. MZ506873.1) and Escherichia phage PC2 (acc. no. NC_073088), was included in the nucleotide comparison (Fig. 1a). Additionally, did we also include Enterobacteria phage Lambda (acc. no. NC_001416.1) as a reference genome, as Enterobacteria phage Lambda shared gene synteny with phages present in the Ymer genus (Fig. 2b). Phages in the Ymer genus exhibited no nucleotide similarity with phage Lambda. However, they did show limited nucleotide similarity with phage KW1E UTAR and phage PC2, with an intergenomic similarity score of 12.2 % and 4.1 %, respectively (Fig. 1a). TEM imaging showed phage Ymer to belong to the *Caudoviricetes* class and displays a siphovirus morphotype (Fig. 1b) (Turner et al., 2023), which is characterized by a flexible, noncontractile tail and an icosahedral geometry of the capsid. The head is non-prolate and displays an average diameter of 53.3 nm (±6.2 nm). Moreover, we determined an average tail length of 161 nm (±9.6 nm) and a tail diameter of 10.7 nm (±0.8 nm). We found evidence for the presence of a central tail fibre but were able to visualize it in only a few TEM images, see Fig. 1b. The structure seems delicate, and it is possible that the fibre broke during phage purification or TEM imaging preparation on some phage particles. No other morphotypes were observed in the TEM images. The genus as a whole is thought to display similar morphotype as phage Ymer, based on the high nucleotide similarity of more than 80 %, however it cannot be excluded that the structure of each phage might differ a bit.

### 3.3 Genomic features shared by the Ymer genus

The genome organization of all seven phages are bi-directional, which is a known feature of phage genomes (Spiegelman et al., 1972). Annotations and genetic structures of the Ymer genus are displayed in Fig. 2. Genes in the Ymer genus can generally be divided into three major groups: ‘Head and packaging’ (pink in Fig. 2a, b), ‘Tail’ (green in Fig. 2a, b) and ‘Nucleotide metabolism’ (purple in Fig. 1a, b). Genes involved in lysis, Endolysin and Rz-like spanin, are found within genes involved in nucleotide metabolism in all seven phage genomes. Rz-like spanin is a two-component system encoded on the genes Rz and Rz1 known from phage Lambda. The Rz and the Rz1 genes are often found together and there coding regions often overlap but in different reading frames (Kongari et al., 2018). In the Ymer genus only Rz was annotated. However, Rz1 is likely overlooked because the gene caller penalizes overlapping genes. One tRNA is found within genes involved in morphogenesis and packaging for all seven phages as well (Fig. 2a, b). No genes were annotated as virulence factors or antimicrobial resistance genes (AMR).

### 3.4 Genomic differences within the Ymer genus

All the seven phages contain a gene annotated as a membrane-associated protein in the same location in the genome (Fig. 2a, b, dark purple star). However, the membrane-associated protein in phage Sabo does not share any amino acid sequence similarity with the membrane-associated proteins in the other six phages, which are identical (Fig. 2a, b, dark purple star). To determine if the two membrane-associated proteins within the Ymer genus may share 3D protein structure, despite no amino acid sequence similarity, ColabFold was used to predict the 3D structure of the two proteins (suppl. fig. 4). A structural alignment by PyMOL of the two membrane-associated proteins resulted in an RSMD value of 2.928 Å. For identical structures the RMSD value should be 0 Å and increases as the two structures become more different. In general, values between 0 and 2 Å reflects high structural similarity. RMSD data calculated for structure pairs of different sizes, as in this case, can however not be directly paired since the RMSD value depends on the number of atoms included in the alignment (Carugo and Pongor, 2001). Both genes shared amino acid sequence similarity with hypothetical proteins found in *Pectobacterium* isolates in the NCBI database (varying from query cover of 98–99 %, and percent identity of 33.6–49.06 %, blastn). Furthermore, the membrane-associated protein shared by Ymer, Amona, Koroua, Pappous, Abuela and Taid also show similarity (query cover: 91 %, percent identity: 38.94 %, blastn) with a gene annotated as a membrane-associated protein in *Pectobacterium* phage DU_PP_V (acc. no. YP_009795315). All phages encode at least one gene encoding DNA methyltransferase (light blue star in Fig. 2a, b), and three out of the seven phages, phage Amona, Sabo and Pappous, encode an additional gene annotated as DNA methyltransferase.

### 3.5 The Ymer genus share genome synteny but not lifestyle with distant relative phage lambda

To find the closest relative to the Ymer genus we did a blastp search against the nr protein database using large terminase and major head protein in phage Ymer (suppl. fig. 3). The closest relative (based on phylogenetic analysis in suppl. fig. 3 as well as intergenomic nucleotide similarity in Fig. 1a) was found to be Escherichia phage KW1E UTAR (acc. no. MZ506873.1). We compared genome structure and sequence similarity of phage KW1E UTAR to the seven Ymer phages (Fig. 2b, Fig. 1a). Furthermore, because of clear similarity in genome organization, Escherichia phage Lambda was also included in our comparisons. It is well known that despite no nucleotide similarity, phage genomes reveal some common genome architectures. In general, phages with siphovirus morphotype share gene synteny between genes encoding virion structure and assembly function (Hatfull, 2008), which also appear obvious between the Ymer genus, phage KW1E and phage Lambda, despite no amino acid sequence similarity shared with phage Lambda for any of the other phages (Fig. 2b ‘Head & Packaging’ pink genes). Likewise, it has been described that in lambda like phages their head genes are upstream of the tail genes (Casjens, 2005; Hatfull, 2008), which also is the case for all phage genomes included in the comparison (Fig. 2a, b, ‘Head &Packaging’ pink genes, ‘Tail’ green genes).

Phage Lambda does however share less synteny among genes encoding DNA metabolism (Fig. 2b ‘DNA metabolism’ light purple genes) and genes involved in lysogeny (Fig. 2b ‘Integration and excision’ light rosa), where the latter is not found in either phage KW1E UTAR or within the Ymer genus (Fig. 2b). The absence of genes involved in lysogeny in both phage KW1E UTAR and within the Ymer genus indicate phages to be strictly lytic, versus phage Lambda which is a temperate phage.

### 3.6 *In-silico* host range predicts the Ymer genus to target three species of Pectobacterium

**Fig. 3:**
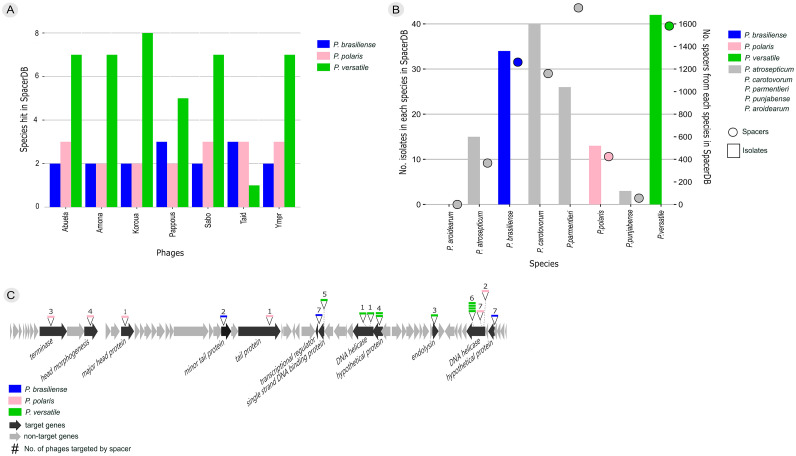
*In silico* host range prediction. a) *In silico* host range prediction using CRISPR spacers. Species hit when aligning phages within the Ymer genus against the CRISPR spacer database SpacerDB, resulting in three *Pectobacterium* species encoding spacer sequences against phages in the Ymer genus (blue; *P. brasiliense*, pink; *P. polaris* and green; *P. versatile)*. b) Number of isolates (bars) as well as number of spacers (circles) within each species known to cause soft rot in potatoes in the SpacerDB (blue; *P. brasiliense*, pink; *P. polaris* and green; *P. versatile,* grey; *P. atrosepticum, P. carotovorum, P. parmentieri, P. punjabense, P. aroidearum)*. c) Mapping CRISPR spacers to compiled consensus genome of the Ymer genus. All spacers were mapped against the Ymer phages and spacer target genes were visualized using a compiled consensus genome of the Ymer genus. Each colored line represents a spacer, the color depends on species as in *a* + *b* (blue; *P. brasiliense*, pink; *P. polaris* and green; *P. versatile)*. Grey arrows represent non-target genes and black arrows represent target genes. Annotation is only shown for target genes. Numbers on top of spacers represent the number of phages within the Ymer genus targeted by the spacer(s).

To investigate phage host range *in silico*, we used a CRISPR spacer database, SpacerDB, using CrisprOpenDB, to get an insight into the bacterial species that harbor CRISPR spacers against the phages within the Ymer genus (Fig. 3a). This showed that *P. brasiliense, P. polaris* and *P. versatile* encoded spacers matching the seven phages in the Ymer genus, with identities ranging from 96 to 100 %. To validate that our results were not due to an overrepresentation of spacer sequences from the three given species, we estimated the number of spacers as well as number of isolates of all *Pectobacterium* species (Fig. 3b), known to cause soft rot (Charkowski, 2018; Curland et al., 2021). The highest numbers of spacers (as well as isolates) in the database were found between *P. carotovorum, P. brasiliense, P. parmentieri* and *P. versatile,* varying from 1100 to 1700, where *P. atrosepticum* and *P. polaris* had 367 and 424 spacers in the database, respectively. Lastly *P. punjabense* only had 56 spacers, and *P. aeroidearum* 0 spacers in the database (Fig. 3b, suppl. Table 3). All spacers were mapped to the phage genomes and visualized in a combined consensus genome of the Ymer genus (Fig. 3c). All spacers do mainly target annotated genes with a known function except two genes annotated as hypothetical proteins. Most of the spacers target sequences within genes present in more than half of the phage genomes, and less of the spacers target sequences within genes present in only a few or even one of the phage genomes (Fig. 3c, suppl. Table 2).

### 3.7 Experimental host range verifies *in silico* host range prediction of the Ymer genus

To validate the *in-silico* host range prediction, we did an experimental host range experiment using soft rot isolates (suppl. Table 1). The initial spot test of all phages showed clearance mainly against *P. brasiliense* but also *P. polaris* and *P. versatile*. Efficiency of plating (EOP) was determined for all phages against all isolation hosts which initially showed clearance in the spot test (Table 2). All phages primarily target *P. brasiliense*; however, phage Abuela and Koroua were also able to infect *P. polaris* and phage Koroua also infected *P. versatile*. Lysis but no plaque formation was observed for several hosts and noted as CZ (clearing zone) in Table 2. To test for any prophage activity all phage host were filtered and included in the initial spot test with all phages. For all filtered phage hosts, two of seven hosts, J21.1 (Taid host) and 5.1 (Pappous host), showed clearing zone but no plaque formation for several *P. brasiliense* isolates and one *P. polaris* isolate (J20, J21, J37, J41 and J19) (data not included in Table 2).

**Table 2:** EOP values for all phages against all bacterial isolates. EOP values were calculated as titer on test host divided by titer on isolation host (noted as 1* for each phage). CZ represents clearing zone but no plaque formation when spotting undiluted phage lysate for each phage. - represents no clearing or plaque formation. Phage titers were as follows, Ymer: 1.3 × 10^9^ PFU/ml, Taid: 2.6 × 10^9^ PFU/ml, Pappous: 1.3 × 10^9^ PFU/ml, Abuela: 3.6 × 10^9^ PFU/ml, Koroua: 8.3 × 10^10^ PFU/ml, Amona: 3.6 × 10^9^ PFU/ml, Sabo: 5 × 10^9^ PFU/ml.

		**Phage EOP**						
**Bacterial ID**	**Taxon**	**Ymer**	**Taid**	**Pappous**	**Abuela**	**Koroua**	**Amona**	**Sabo**
J13	*P. brasiliense*	0.97	1.17	1*	0.45	6.69E-04	0.01	CZ
J20	*P. brasiliense*	0.05	CZ	CZ	0.01	CZ	CZ	–
J21	*P. brasiliense*	0.01	CZ	CZ	0.01	CZ	CZ	–
J24	*P. brasiliense*	0.82	2.40E-04	4.51E-03	1.47	0.03	0.75	1*
J25	*P. brasiliense*	0.97	2.29E-04	6.64E-04	0.73	0.02	1*	1.86
J26	*P. brasiliense*	0.51	1.27E-03	2.48E-03	1.00	0.03	1.00	–
J31	*P. brasiliense*	CZ	1*	9.77E-04	1.20E-04	CZ	CZ	CZ
J32	*P. brasiliense*	0.56	CZ	4.51E-05	1.47	0.03	0.93	2.00
J34	*P. brasiliense*	0.64	6.15E-06	1.85	1*	0.03	0.93	1.86
J36	*P. brasiliense*	1*	3.31E-05	2.26E-05	1.47	0.02	0.93	0.72
J37	*P. brasiliense*	0.05	CZ	1.95E-06	0.25	2.42E-03	0.11	0.02
J41	*P. brasiliense*	3.08E-03	0.54	0.62	0.50	6.05E-04	0.01	–
J44	*P. brasiliense*	0.43	3.96E-05	2.50E-05	0.56	0.02	0.45	–
J45	*P. brasiliense*	–	–	–	–	–	–	–
J47	*P. brasiliense*	0.66	CZ	3.76E-05	0.58	0.01	0.83	1.33
J52	*P. brasiliense*	0.43	CZ	3.98E-05	0.56	1*	0.44	1.33
J59	*P. brasiliense*	1.00E-03	–	CZ	0.01	CZ	CZ	–
J54	*P. versatile*	CZ	–	–	CZ	0.99	CZ	CZ
J17	*P. polaris*	–	–	–	CZ	–	–	CZ
J18	*P. polaris*	–	–	–	–	–	–	CZ
J19	*P. polaris*	–	–	–	0.01	2.42E-04	–	–
J48	*P. polaris*	–	–	–	–	–	–	–
J27	*P. parmentieri*	–	–	–	–	–	–	–
J29	*P. parmentieri*	–	–	–	–	–	–	–
J30	*P. parmentieri*	–	–	–	–	–	–	–
J42	*P. parmentieri*	–	–	–	–	–	–	–
J43	*P. parmentieri*	–	–	–	–	–	–	–
J49	*P. parmentieri*	–	–	–	–	–	–	–
J58	*P. parmentieri*	–	–	–	–	–	–	–
J2	*P. punjabense*	–	–	–	–	–	–	–
J3	*P. punjabense*	–	–	–	–	–	–	–
J56	*P. atrosepticum*	–	–	–	–	–	–	–
J28	*P. atrosepticum*	–	–	–	–	–	–	–
J6	*P. atrosepticum*	–	–	–	–	–	–	–
J7	*P. atrosepticum*	–	–	–	–	–	–	–
J8	*P. atrosepticum*	–	–	–	–	–	–	–
J9	*P. atrosepticum*	–	–	–	–	–	–	–
J10	*P. atrosepticum*	–	–	–	–	–	–	–
J11	*P. atrosepticum*	–	–	–	–	–	–	–
J12	*P. atrosepticum*	–	–	–	–	–	–	–
J16	*P. atrosepticum*	–	–	–	–	–	–	–
J22	*P. atrosepticum*	–	–	–	–	–	–	–
J33	*P. atrosepticum*	–	–	–	–	–	–	–
J38	*P. atrosepticum*	–	–	–	–	–	–	–
J39	*P. atrosepticum*	–	–	–	–	–	–	–
J40	*P. atrosepticum*	–	–	–	–	–	–	–
J35	*Dickeya solani*	–	–	–	–	–	–	–

## 4 Discussion

The genomic characterization of all phages in the Ymer genus showed shared gene synteny, with most of them starting with the gene encoding a terminase followed by genes encoding head morphogenesis and packaging, tail genes and DNA metabolism genes. Gene synteny was also shared between the Ymer genus and phage Lambda and phage KW1E UTAR. The Ymer genus did share both gene synteny and several amino acid sequence similarities with phage KW1E UTAR, where phage Lambda only shared amino acid sequence similarity with two genes (minor tail protein and tail assembly protein) to the genome of the rest of the phages. Despite very little amino acid sequence similarity, the gene synteny were very well preserved, which is a known feature in phage genomics (Hatfull, 2008). Phage genomes are mosaic, but not all genes participate in mosaicism at the same level (Hatfull and Hendrix, 2011), which is seen in the conserved gene synteny shared between all the phages. Interestingly, the shared gene synteny is despite any, or very little, nucleotide similarity between phage Lambda and phage KW1E UTAR and the rest of the Ymer genus. All phages in the Ymer genus did in general show very limited nucleotide similarity to any entries present in the GenBank database. Nonetheless, the only hits were to *Pectobacterium* and *Yersinia* bacterial strains. In most cases we did see a match to genes annotated as a minor tail protein, which can be due to the presence of prophages in the bacterial genomes, and to genes encoding tRNA's. All phages within the Ymer genus encoded one tRNA. It is widely known that many phages encode their own tRNA, which previously has been suggested as a fitness advantage, as the tRNA encoded by the phage genome would correspond to codons which are highly used by the phage genes, but rarely by the host genome (Bailly-Bechet et al., 2007). However, a new preprint by Burman et al. has proved some phages encoded tRNA's to be anti-phage defense systems, as the bacterial phage defense system will trigger translational arrest upon infection by degrading host tRNA. Burman et al. showed that some phages will circumvent this event by encoding non-cleavable tRNA (Burman et al., 2024). GC content in all phages tends to be ∼6 % lower than their isolation host. This fits very well with the fact that on average, phages have ∼4 % higher AT content, with the hypothesis of a bias towards a higher AT content allowing better exploitation of the host cell machinery and that AT-rich DNA is more flexible and thus easier to pack, where the AT content variation in temperate phages seems to be lower than in virulent phages (Bong et al., 2023; Rocha and Danchin, 2002). Even though the Ymer genus shared gene synteny with phage Lambda, which is a temperate phage, none of the phages encoded any genes annotated as integrases or other genes associated with lysogeny. The Ymer genus and phage Lambda did also mainly share gene synteny within ‘Head and Packaging’ and ‘Tail’ genes, where genes involved in lysogeny and integration, in phage Lambda, mainly are found within genes encoding ‘Nucleotide Metabolism’. The Ymer genus also did not encode any genes annotated as virulence factors or antimicrobial resistance.

All phages within the Ymer genus encoded a gene annotated as a membrane-associated protein. The seven phages did, however, not encode the same membrane-associated protein, as phage Sabo encodes a different gene. The 3D structures of the two proteins were aligned, resulting in a RMSD value of 2.928 Å (RMSD value of 0–2 Å indicate similar structure). The proteins do however differ in size, making the value less significant, as the RMSD value depends on the number of atoms included in the structural alignment (Carugo and Pongor, 2001). Both genes annotated as membrane-associated proteins do also share amino acid sequence similarity with *Pectobacterium* proteins in the NCBI database (query cover: 98–99 %, percent identity: 33.6–49.06 %). Interestingly, phage Lambda also encodes a membrane protein (‘LOM outer membrane protein’) at the same gene placement as the gene encoding a membrane associated protein in the Ymer genus, within the Tail genes. This protein has been found to play a role in *E. coli* adhesion to human buccal epithelial cells (Vica Pacheco et al., 1997). The gene synteny between the ‘LOM outer membrane protein’ in phage Lambda and the membrane-associated protein in the Ymer genus indicate some kind of conservation, the protein in phage Lambda is however only active when the phage is integrated as a lysogen of *E. coli*. If the function of this gene is conserved between the Ymer genus and phage Lambda, this would suggest phage Ymer to be temperate as well. Nevertheless, given the fact that the Ymer genus did not encode any genes involved in lysogeny and integration and that the membrane-associated proteins encoded by the genus only show amino acid sequence similarity to *Pectobacterium* proteins annotated as hypothetical, the function of these genes remains a puzzle. It is however interesting that several versions of this gene appear to exist within the genus, and they are usually associated with tail proteins, suggesting that they may be involved in the latter steps of phage replication or host cell lysis.

Several methods are used to determine host range for phages, some being laboratory based using spot test and EOP (Fong et al., 2021), but newer approaches like metagenomic studies link viral genomes to their host based on genomic data (de Jonge et al., 2019). Here we performed an *in silico* host range prediction, but did also use laboratory-based experiments using spot test and EOP. The *in silico* host range prediction for the phages within the Ymer genus resulted in the identification of three *Pectobacterium* host species, i.e. *P. brasiliense, P. versatile* and *P. polaris* (Fig. 3). To predict the *in silico* host range, we used a CRISPR spacer database to align spacers to phage genomes, which indicate how some isolates within these species have acquired CRISPR spacers and thereby immunity against phages encoding target sequences (Fineran and Charpentier, 2012). We did however observe that only one isolate of *P. brasiliense* encoded all three spacers targeted the Ymer genus (suppl. Table 2), which indicates that only one out of 34 isolates (Fig. 3b, suppl. Table 3) present in the database have acquired CRISPR spacers against phages within the Ymer genus. Likewise, only 8 of 42 isolates of *P. versatile* and three of 13 isolates of *P. polaris* encoded all CRISPR spacers from the given species, targeting phages within the Ymer genus (suppl. Table 2, suppl. Table 3). This gives us an idea that most isolates in the database within these three species have not acquired CRISPR spacer sequences targeting phages within the Ymer genus, but do however indicate which species which may be a potential host for these phages. Interestingly, the spacers primarily target annotated genes (only 2 out of 12 genes were annotated as hypothetical protein). It is a known feature that CRISPR spacers targets are unevenly distributed, which suggests a strong selection on specific phage genes (Heler et al., 2019; Paez-Espino et al., 2013). Likewise, has it been shown that spacers may target several different phages if target site is placed within a conserved phage region (Walker and Shields, 2022). In accordance with this we did also see all spacers, despite the two spacers targeting hypothetical proteins, target phage genes annotated with essential functions (Kristensen et al., 2011). These included both structural proteins (head and tail genes), DNA replication (terminase, transcriptional regulator, DNA binding protein, DNA helicases) and one spacer targeted endolysin, involved in lysis of the cell. As most of these genes are known to be conserved phage regions, spacers may be selected for as these may target other phages encoding the same regions. To evaluate whether our *in-silico* host range prediction was due to an overrepresentation of spacer sequences in the database from the three given species, we estimated the number of spacer sequences of all *Pectobacterium* species known to be causing soft rot in potatoes (Fig. 3b, suppl. Table 3). As our result did not correlate with all dominating species in the database (dominating species: *P. carotovorum, P. brasiliense, P. parmentieri, P. versatile*), we concluded that our result was not due to an overrepresentation of spacer sequences in the database of the three given species. Interestingly, the experimental host range result verified the host range potential for the Ymer genus as a whole, as the phages all together were able to infect isolates from the three *Pectobacterium* species *P. brasiliense, P. versatile* and *P. polaris*. The host-range experiment showed that two phages (Abuela and Koroua) of the seven, were able to infect more than one species of *Pectobacterium,* where phage Koroua were able to infect three species of *Pectobacterium*, being *P. brasiliense, P. versatile* and *P. polaris*. To further elaborate on the biocontrol potential, it would have been of interest to estimate the growth rate of the phages using different MOI's (multiplicity of infection) as this would give an indication of their effectiveness. Likewise, it would have been interesting to compare mixed cultures of phages versus monocultures. Phage cocktails are often referred to as more effective as these may inhibit the emergence of phage resistance and may be able to target more broadly (Abedon et al., 2021). To screen for any prophage activity, overnight cultures of all phage hosts were filtered, and the filtrate used for the initial spot test in the host range. Two of the filtered phage hosts did show clearance but no plaque formation. This phenomenon could be due to prophage activity or toxins originating from these host filtrates. However, if this activity is indeed linked to prophages, it is evident that these prophages were unable to replicate sufficiently to form visible plaques.

## 5 Conclusion

The seven phages in the Ymer genus displayed similar genome synteny with distantly related phage KW1E UTAR and phage Lambda, and despite their genome similarity the Ymer genus did reveal differences within genes annotated as both membrane-associated proteins and DNA methyltransferases, among others. None of the phages encoded any integrases or other proteins involved in lysogeny and nor did they share gene synteny with genes involved in lysogeny in phage Lambda, which could suggest that these phages are strictly lytic. An *in-silico* host range determination of the Ymer genus predicted both *P. brasiliense, P. polaris* and *P. versatile* as potential hosts and the experimental host range of the Ymer genus as a group verified this potential. In an applied perspective both phage host range and lifestyle should be considered, where it is often suggested that phages used as biocontrol should be strictly lytic. These phages could be valuable as biocontrol agents both from a point of view of the phages being strictly lytic and more importantly their host range, which include soft rot isolates from Danish tubers and plants.

## Declaration of competing interest

The authors declare that they have no known competing financial interests or personal relationships that could have appeared to influence the work reported in this paper.

## Appendix Supplementary materials

Image, application 1

Image, image 2

Image, image 3

Image, image 4

Image, image 5

Image, application 6

Image, application 7

Image, application 8
