# Supplementary material for: A novel genus of Pectobacterium bacteriophages display broad host range by targeting several species of Danish soft rot isolates
Source: Virus Res. 2024 Jul 16;347:199435. doi: 10.1016/j.virusres.2024.199435 (PMC11445585; doi:10.1016/j.virusres.2024.199435)
Supplement: Supplementary file 1 [file mmc1.docx]

**Supplementary figure 1.** Sequence coverage around the termini position plotted by Phageterm for phage Pappous (a) and phage Amona (b). Termini positions are represented by the red line. For both phages Phageterm predicted their packaging strategy being categorized as headful packaging, which can be seen as higher coverage after the packaging site (Garneau et al., 2017). Phageterm was not able to predict startsite for any of the other phages within the Ymer genus.

**Supplementary figure 2.** Phylogenetic analysis using 16S rRNA gene sequences from bacterial isolates and reference sequences (RefSeq) in the NCBI database. Reference sequences include known species causing soft rot in potatoes. All isolates are color ranged based on closest relative on species level; *Dickeya solani* (pink), *P. parmentieri* (yellow), *P. atrosepticum* (green), *P. punjabense* (grey), *P. versatile* (blue), *P. polaris* (purple) and *P. brasiliense* (orange). All reference sequences used for 16S rRNA gene phylogenetic analysis are in bold and are as follows; *Dickeya dadantii* (RefSeq ID: NC_014500.1), *Dickeya dianthicola* (RefSeq ID: NZ_CP031560.1), *Dickeya solani* (RefSeq ID: NZ_CP017454.1), *Dickeya chrysanthemi* (RefSeq ID: NC_012912.1), *P. parmentieri* (RefSeq ID: NZ_CP027260.1), *P. atrosepticum* (RefSeq ID: NZ_CP009125.1), *P. aroidearum* (RefSeq ID: NZ_CP065044.1), *P. punjabense* (RefSeq ID: NZ_CP038498.1), *P. carotovorum* (RefSeq ID: NZ_CP051652.1), *P. versatile* (NZ_CP021894.1), *P. polaris* (RefSeq ID: NZ_CP017482.1), *P. brasiliense* (RefSeq ID: NZ_CP047495.1).

**Supplementary figure 3.** Phylogenetic analysis of the Ymer genus (green group) using major head protein (a) and large terminase (b) in phage Ymer. Closest relative based on the protein sequences of large terminase (orange group) and major head protein (blue group) in phage Ymer using blastp were included in each phylogenetic analysis. Large terminase and major head protein were subtracted from each phage and included in each phylogenetic analysis. Phages included in the phylogenetic analyses were as follows; Escherichia phage KW1E UTAR (acc. no. MZ506873), Serratia phage vB SmaM-Susuwatari (acc. no. ON287371), Serratia phage vB SmaM-ChibiTotoro (acc. no. ON287368), Klebsiella phage P85_2 (acc. no. OR256025), Klebsiella phage vB Kpn K34PH164 (acc. no. OY979427), Escherichia phage PC2 (acc. no. NC_073088), Enterobacteria phage EK99P-1 (acc. no. NC_024783), Escherichia phage vB EcoD Teewinot (acc. no. NC_073053), Escherichia phage slur05 (acc. no. NC_028901), Escherichia phage vB_EcoS-EE09 (acc. no. OR756193). Furthermore, did we include Enterobacteria phage Lambda (acc. no. NC_001416.1) as reference genome. Genuses are added for each phage group (if assigned to any). All phage isolates being closest relative to the Ymer genus based on major head protein (blue) are all part of the genus: *Dhillonvirus.* No genus was assigned to any of the phage isolates being closest relative to the Ymer genus based on large terminase (orange).

**Supplementary figure 4.** Alignment of membrane-associated proteins 3D structure (RMSD = 2.928 Å). 1) membrane associated protein found in phage Sabo. 2) membrane-associated protein found in phage Ymer, Amona, Koroua, Pappous, Abuela and Taid. Both membrane proteins are marked with purple stars in figure 2.

**Supplementary table 1.** Bacterial isolates used for the host range initial spot test. All host are classified with Genbank accession numbers, and phage isolation hosts are classifies with phage name.

**Supplementary table 2.** Overview of spacer sequences targeting phages within the Ymer genus, including; spacer ID (in SpacerDB), species, strain name (NCBI), spacer ID, target gene, target phage (Dion et al., 2021b).

**Supplementary table 3.** Number of isolates in database as well as number of spacer sequences from each *Pectobacterium* species in the CRISPRopendb (Dion et al., 2021b).
