# Supplementary figures and images for: A novel genus of Pectobacterium bacteriophages display broad host range by targeting several species of Danish soft rot isolates
Source: Virus Res. 2024 Jul 16;347:199435. doi: 10.1016/j.virusres.2024.199435 (PMC11445585; doi:10.1016/j.virusres.2024.199435)

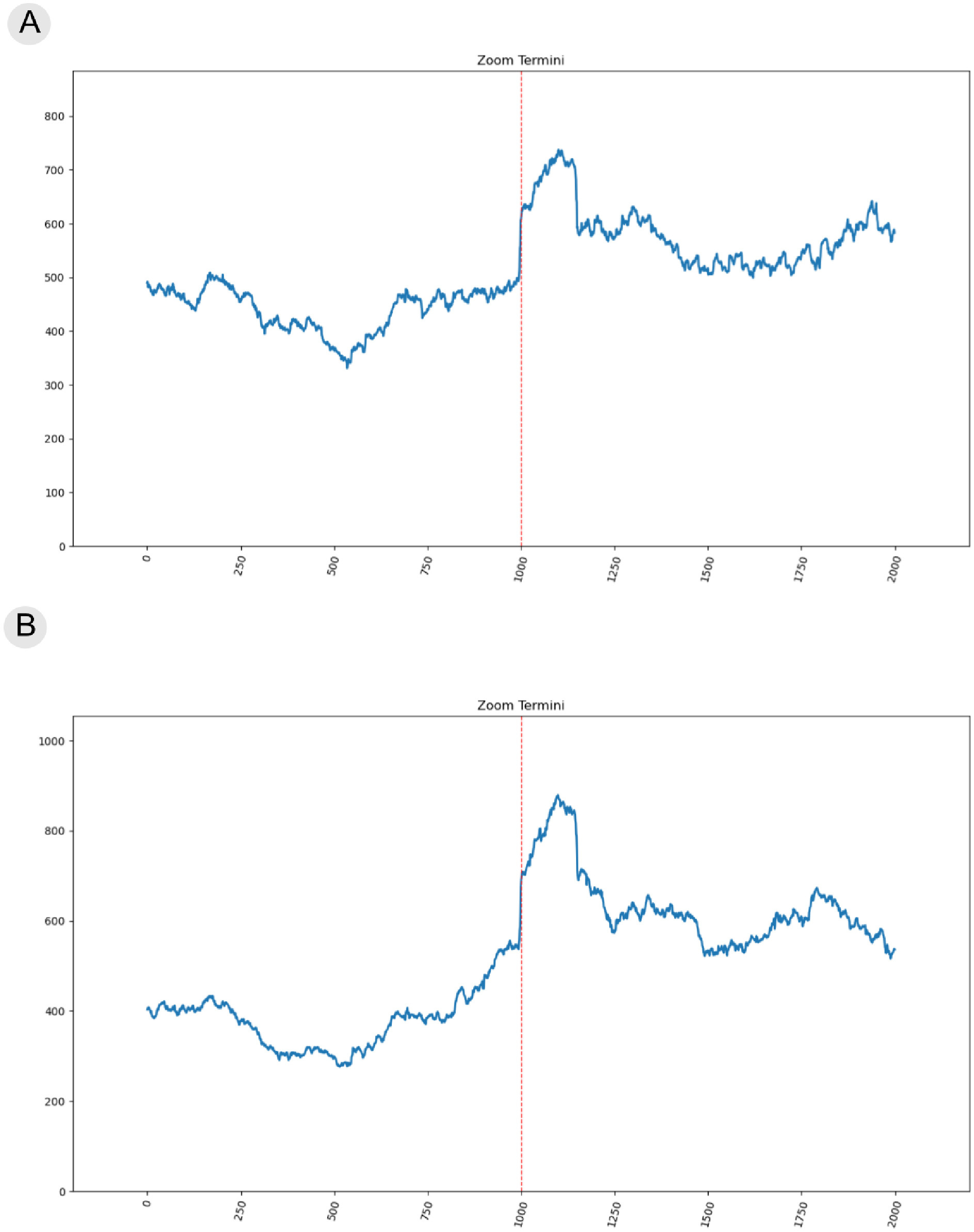

Supplement: Supplementary file 2 [file mmc2.jpg]

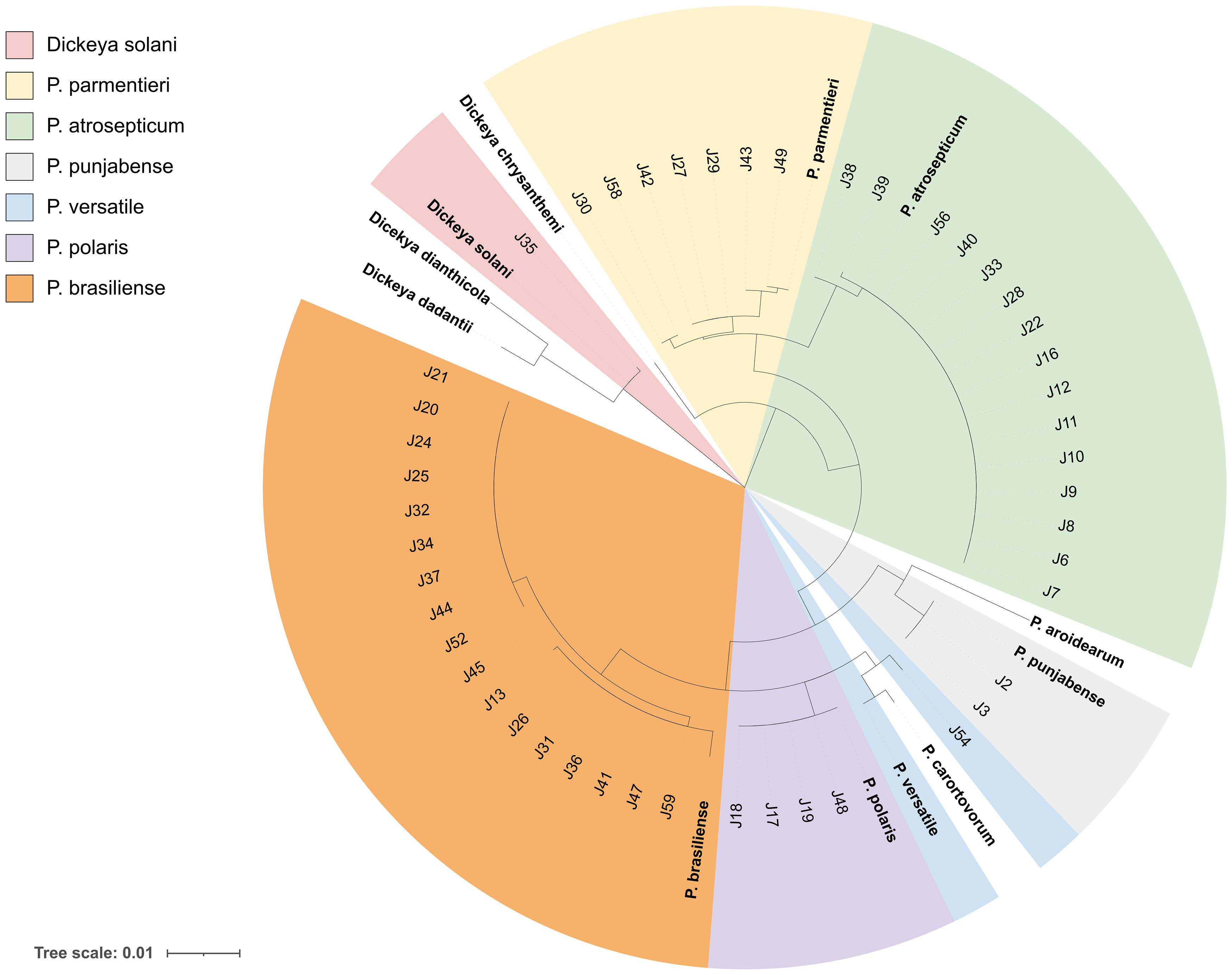

Supplement: Supplementary file 3 [file mmc3.jpg]

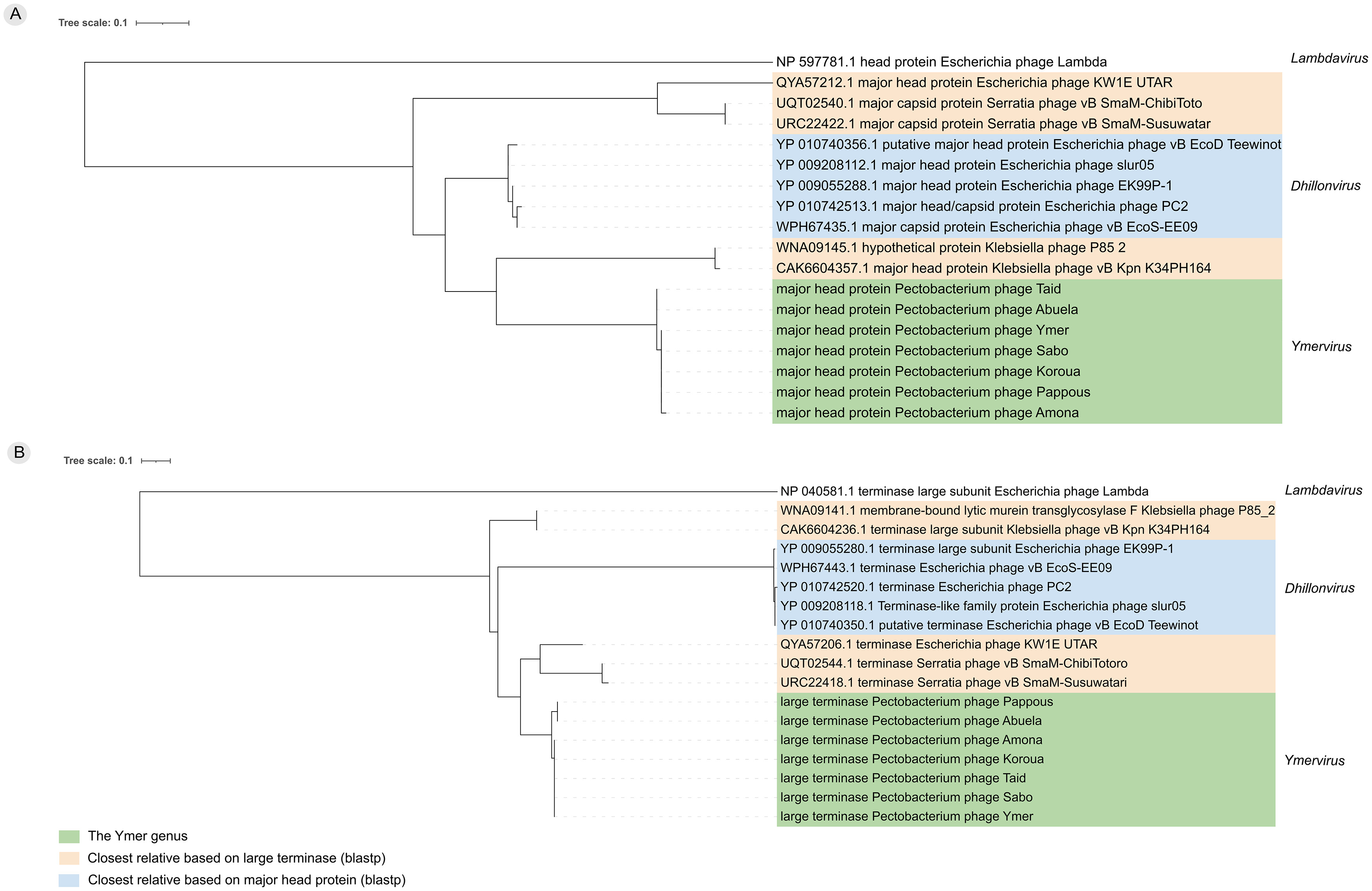

Supplement: Supplementary file 4 [file mmc4.jpg]

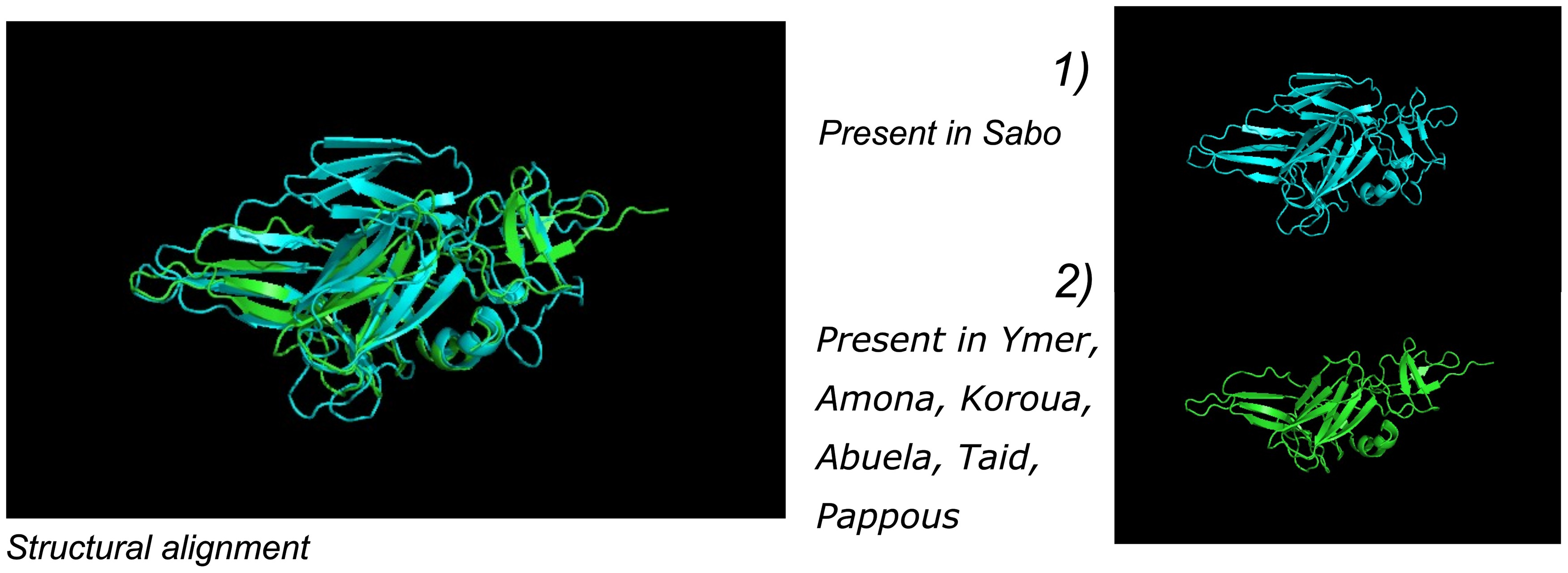

Supplement: Supplementary file 5 [file mmc5.jpg]
